# Understanding and Addressing COVID-19 Vaccine Hesitancy Among Healthcare Providers in Bexar County, Texas

**DOI:** 10.1016/j.focus.2022.100022

**Published:** 2022-08-10

**Authors:** Hari N. Krishnakumar, Jay H. Shah, Lucas S. Rivas, Jason A. Rosenfeld, Courtney G. Denton, Melanie Stone, Anita Kurian, Ruth E. Berggren

**Affiliations:** 1 Center for Medical Humanities & Ethics, The University of Texas Health Science Center San Antonio, San Antonio, Texas; 2 San Antonio Metropolitan Health District, San Antonio, Texas

**Keywords:** COVID-19, vaccine, vaccine hesitancy, healthcare providers

## Abstract

**Introduction:**

COVID-19 challenged our healthcare systems and unsurprisingly, so did its vaccine. Chief among these were the uniquely politicized nature and logistical difficulties surrounding its deployment. Understanding provider attitudes toward the COVID-19 vaccines and their willingness/ability to deliver them is essential to developing an ideal vaccine distribution plan for Bexar County, Texas.

**Methods:**

An electronic survey was sent to providers serving adult patients across Bexar County. Membership includes representation from local hospitals, Bexar County Medical Society, and the San Antonio Metropolitan Health District's vaccination program. The survey measured provider attitudes toward the vaccines and asked logistical questions regarding patient hesitancy and clinic infrastructure.

**Results:**

Responses were collected from 66 ZIP codes (90% of ZIP codes in the county), and 377 providers answered the survey. Respondents who were unlikely to recommend the vaccine (3%) shared concerns involving distrust of the vaccine manufacturing process. Recommendations made by physicians to support their vaccine administration efforts included broadening the reporting timeframe (*n*=35), providing trained support staff (*n*=18), and improving logistical support (*n*=14). Furthermore, responses showed that only 14.44% of provider facilities were able to meet originally published refrigeration requirements to store Pfizer's vaccines compared with 87.47% for Moderna's vaccines.

**Conclusions:**

Survey results were used to improve the efficiency of vaccination programs and address vaccine hesitancy through various avenues of information delivery. It is recommended to replicate the outcomes and applications of this study in other populations to improve the efficiency of vaccination programs and reduce vaccine hesitancy.

## HIGHLIGHTS

• More than 35% of responding physicians had concerns regarding the vaccine.
• A total of 20% of respondents supported vaccination under Food & Drug Administration's full approval.
• A total of 77% of respondents supported an emergency use authorization.
• Data aided county-wide vaccination by identifying regions lacking vaccine providers.

## INTRODUCTION

As of June 24, 2022, WHO reports more than 542 million confirmed cases of the novel virus severe acute respiratory syndrome-coronavirus 2 (SARS-CoV-2) globally and nearly 86.8 million within the U.S. alone.^1^ Texas reported more than 7 million cases at the time of writing and a 7-day moving average of new cases in Texas ranging from 17,000 to peaking at nearly 23,000 cases.^2^ Bexar County has the fourth highest number of cases in Texas with an estimated 588,000 cases. The number of hospitalizations and deaths followed similar patterns. Mass access to vaccines took on the utmost importance to curb the spread of the pandemic, and years of research and development ushered in new technology in the form of messenger RNA vaccines to reduce the spread and mortality rate of this virus.^3^ However, significant barriers still remained, ranging from the hesitancy among patients and healthcare providers and complications regarding logistics and vaccine distribution.

Even before their release, there were concerns among some healthcare providers about vaccine safety and efficacy.^4^ Furthermore, the uniquely politicized nature of the pandemic and the speed at which vaccines were approved for emergency use by the Food and Drug Administration (FDA) reduced public confidence.^5^^,^^6^ This subgroup of the provider population who were unsure about the vaccine was broad reaching. In fact, it was estimated that 31% of the physicians were unsure about receiving the vaccine, albeit in mid-September 2020, during the early stages of vaccine discussion.^7^ In addition to these concerns, there were also logistical issues relating to storage and distribution, which further complicated the vaccine rollout plan.

The fact that vaccines are an important part of any prevention effort is of consensus among healthcare workers, and thus, the challenge was to tackle any causes for hesitancy immediately. Throughout the years, physicians and other healthcare providers dominate most trusted professions lists, and this trend has continued throughout the pandemic. The importance of the longstanding relationships many providers have with their patients cannot be understated as reflected in a monthly survey published by the Delphi Group at Carnegie Mellon University in partnership with Facebook. Results showed that vaccine-related messaging through local healthcare workers was more successful in encouraging vaccine-hesitant populations than other information sources. Despite this, San Antonio has a unique challenge because of its prominent racial and ethnic minority population, given that vaccine hesitancy is suggested to be higher among racial and ethnic minorities.^8^ Trust has been lost among racial and ethnic minorities because of a history of discrimination, unethical healthcare research in Black populations, and negative experiences in a culturally unaccommodating healthcare system.^9^ In addition, vaccine distribution efforts often tend to overlook racial and ethnic minority populations that lack access to or are remote to larger vaccine distribution hubs.^10^

A survey to ascertain sentiment also allows for the opportunity to check for potential logistical or auxiliary concerns that can then be reported to the local health department San Antonio Metropolitan Health District (SAMHD) to inform their action plan for rollout. An important avenue to address vaccine hesitancy at large would be to understand sources of hesitancy from the healthcare provider's perspective. Tackling it at this level and addressing the concerns within our community's healthcare providers would improve their confidence in the vaccine and likely foster a more positive outlook, effects which would be seen downstream in their patient's beliefs and opinions about the vaccine.

## METHODS

This study used a cross-sectional design with an electronic survey. This survey was developed using existing tools that assessed health provider attitudes toward immunization programs and newer measures specific to coronavirus disease 2019 (COVID-19) vaccines. Owing to the emergent need for this study to inform the design of vaccine programs, a purposive sample of physicians and public health professionals supporting Bexar County's COVID-19 response reviewed the survey for question clarity and usefulness before the study launch. Surveyed measures were reviewed by an interprofessional team of public health professionals, physicians, and vaccine communication experts.

### Study Population

The survey was disseminated to internal medicine, family medicine, and community medicine physicians working across Bexar County. Physicians were recruited using listservs managed by the Bexar County Medical Society (BCMS), the SAMHD's Vaccines for Children and Adult Safety Net programs, and the South Texas Regional Advisory Council (STRAC).

### Measures

The survey was open throughout November 2020 and measured provider attitudes toward the COVID-19 vaccines; the common information sources physician used for vaccine information; and the perceived barriers to successful vaccine uptake and distribution, including logistical barriers, such as insurance coverage, patient hesitancy, and clinic infrastructure. Demographic information was also collected and analyzed regarding sex, ethnicity, race, and number of years practiced after residency or other training.

### Statistical Analysis

The survey was developed, deployed, and analyzed through the REDCap tool. Responses to close-ended questions were analyzed quantitatively to include frequencies and mean/median responses, whereas responses to open-ended questions underwent content analysis to develop descriptive statistics. Correlations between the frequency of responses by ZIP codes and COVID-19 case rates and also the COVID-19‒related fatality rates were analyzed for trends. The study was approved by the UT Health San Antonio IRB as exempt research under protocols 20–830E.

## RESULTS

The results of the survey encompassed a diverse array of perspectives from healthcare providers across Bexar County. Responses were collected from 377 providers working in 66 ZIP codes across Bexar County (90% of ZIP codes in the county). Demographic distributions of respondents were collected by sex, ethnicity, and race (Table 1). There was a nearly even ratio of male-to-female respondents, and nearly 32% of respondents were of Hispanic or Latino origin. When looking at the racial distribution, a strong majority of respondents identified as White or Caucasian. More than half of respondents had been practicing for >20 years (53.3%), whereas 25% had been practicing for 10–20 years (25.2%), and 16% had been practicing for <10 years. In addition, the patient populations cared for by the sampled providers encompassed a diverse set of groups ranging from veterans to the homeless (Table 2).

**Table 1:** Summary of Respondent Demographics

Provider characteristics	Frequencies, *n*	Percentages, %
Sex		
Male	144	51.80
Female	133	47.84
Prefer not to answer	1	0.36
Total	278	100.00
Ethnicity		
Not Hispanic or Latino	177	64.13
Hispanic or Latino	88	31.88
Do not wish to answer	8	2.90
Unknown/not reported	3	1.09
Total	276	100.00
Race		
White or Caucasian	231	83.09
Asian	17	6.12
Do not wish to answer	12	4.32
More than one	8	2.88
Black or African American	6	2.16
Native American/Alaska Native	2	0.72
Unknown/other	2	0.72
Total	278	100.00

*Note:* Responses to questions on demographics in Table 1 were optional.

**Table 2:** Summary of Respondent's Patient Populations

Patient population characteristics	Frequencies, *n*	Percentages, %
Racial and ethnic minority groups	328	91.88
Adults aged 19–64 years	283	78.18
Adults aged ≥65 years	279	77.29
People who are underinsured or uninsured	274	77.18
Healthcare workers	259	72.55
Military, veteran	217	61.47
Children aged ≤18 years	204	56.67
Pregnant women	152	43.06
Long-term care facility residents (nursing home, assisted living, or independent living facility)	141	40.06
People experiencing homelessness	134	38.40
Military, active duty/reserves	131	37.43
Medicaid participants	267	71.01
VFC participants	83	22.07
ASN participants	26	6.91

*Note:* Responses to questions regarding provider's patient populations in Table 2 were optional.

ASN, Adult Safety Net; VFC, Vaccines for Children.

Nearly 7% of providers were enrolled in Adult Safety Net, and 22% of providers were enrolled in Vaccines for Children vaccination programs, whereas more than 70% accepted Medicaid (Table 2). Respondents worked at Federally Qualified Health Centers, government hospitals, long-term care centers, occupational health clinics, urgent care/emergency care centers, private hospitals, private primary care clinics, and private specialty care clinics.

Most physicians relied on peer-reviewed references, such as published clinical trial data (*n*=319, 84.62%) and information from government agencies such as the Centers for Disease Control and Prevention (*n*=317, 84.08%), as their primary source of COVID-19 vaccine information. Other sources included public health leaders (*n*=264, 70.02%) such as Anthony Fauci and the WHO, physician networks (*n*=230, 61.00%) such as the American Medical Association and Texas Medical Association, and personal experiences with vaccines and COVID-19 (*n*=116, 30.76%). Less frequently cited sources of information by respondents included pharmaceutical companies (*n*=64, 16.98%), nongovernmental agencies (*n*=31, 8.22%), and social media (*n*=34, 9.02%).

**Figure 1:**
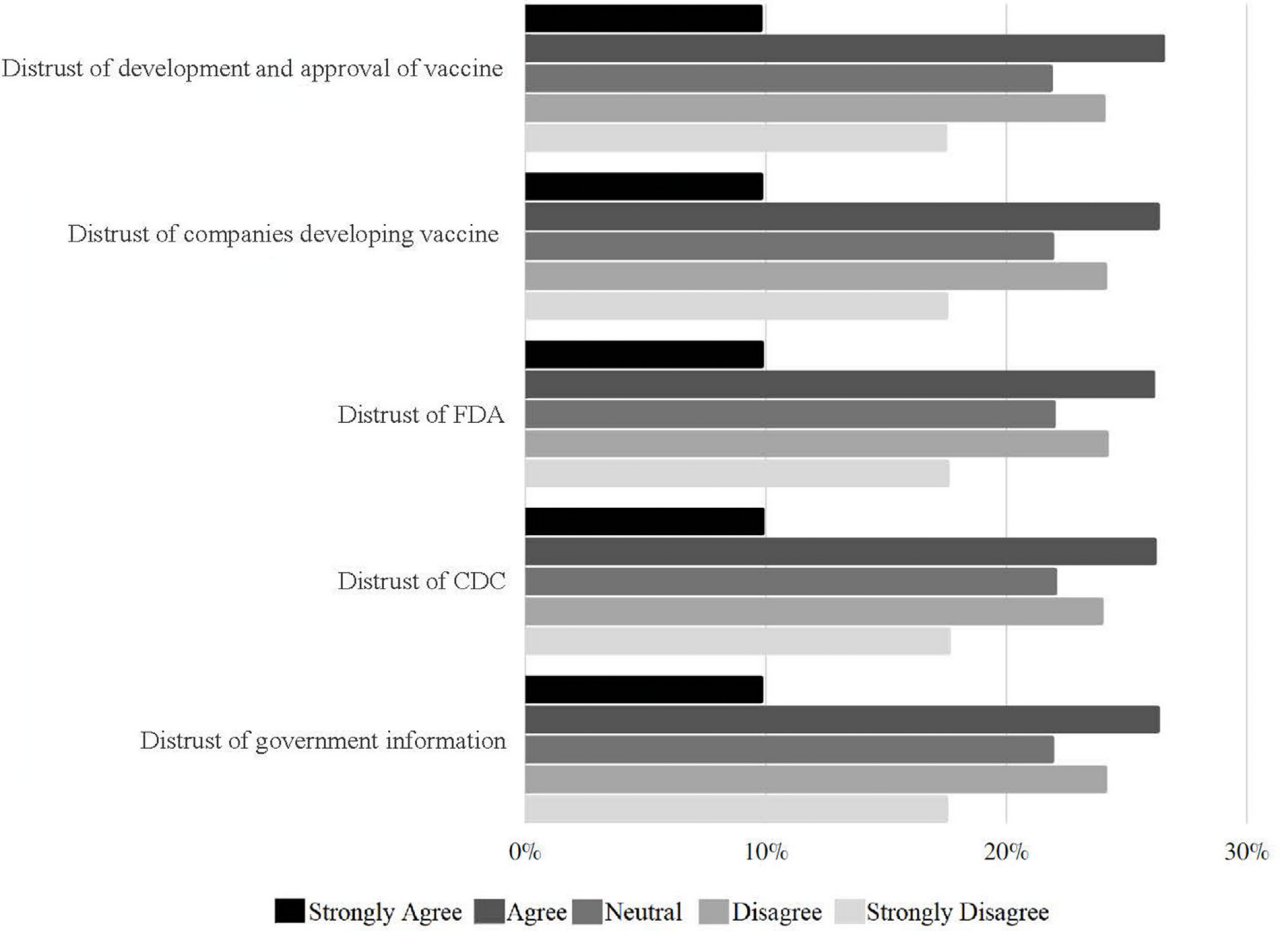
Summary of respondent's fears and concerns about COVID-19 vaccines. Of 377 respondents, 367 chose to respond to questions regarding each of these items. This figure shows the percentage distribution of responses to each item of the 367 total responses.

CDC, Centers for Disease Control and Prevention; FDA, Food and Drug Administration.

More than 35% of responding physicians had concerns regarding the vaccine (Figure 1). These fears are related to distrust of pharmaceutical companies developing and manufacturing the vaccine and government agencies such as the FDA and Centers for Disease Control and Prevention overseeing vaccine development. There is also distrust regarding the information provided by the federal government about COVID-19 and its vaccines, with many questioning the rapid approval of the vaccine. Other concerns surrounding the vaccine include its politicized nature as well as the lack of information regarding long-term effects.

When asked about vaccine storage facilities, responses were collected from 367 participants. A total of 12.53% (*n*=46) reported that they or their organization did not have the facilities to store vaccines at 2‒8 °C. In addition, during the earlier stages of distribution and vaccination, when it was assumed that Pfizer must be stored at −60 to −80 °C, it was seen that upward of 85.56% (*n*=314) of providers would not be able to store at these temperatures.

At the time of data collection, nearly 20% of respondents supported administering vaccines under FDA full licensure approval, and 77% supported an emergency use authorization (EUA), especially for patients with multiple comorbidities. Respondents stated that they would support or further their existing support regarding vaccinations with more robust safety and efficacy data (*n*=184, 50.1%), worsening regional outbreaks (*n*=156, 42.5%), or broader acceptance publicly (*n*=87, 23.7%) and in the healthcare community (*n*=191, 52.0%).

When asked how the SAMHD could help to address vaccine hesitancies, the most common suggestion was to implement vaccine education programs (*n*=135, 38.68%). Providing clear and concise information (*n*=102, 29.23%) on vaccine research, especially regarding vaccine safety, was also cited as a way to combat vaccine hesitancy. Additional vaccine research (*n*=34, 9.74%), especially research in vaccine safety (*n*=31, 8.88%), was also cited as having a positive impact on alleviating reasons for vaccine hesitancy, along with offering public endorsements of the vaccine (*n*=16, 4.58%) and increasing the accessibility of vaccines (*n*=13, 3.72%).

**Figure 2:**
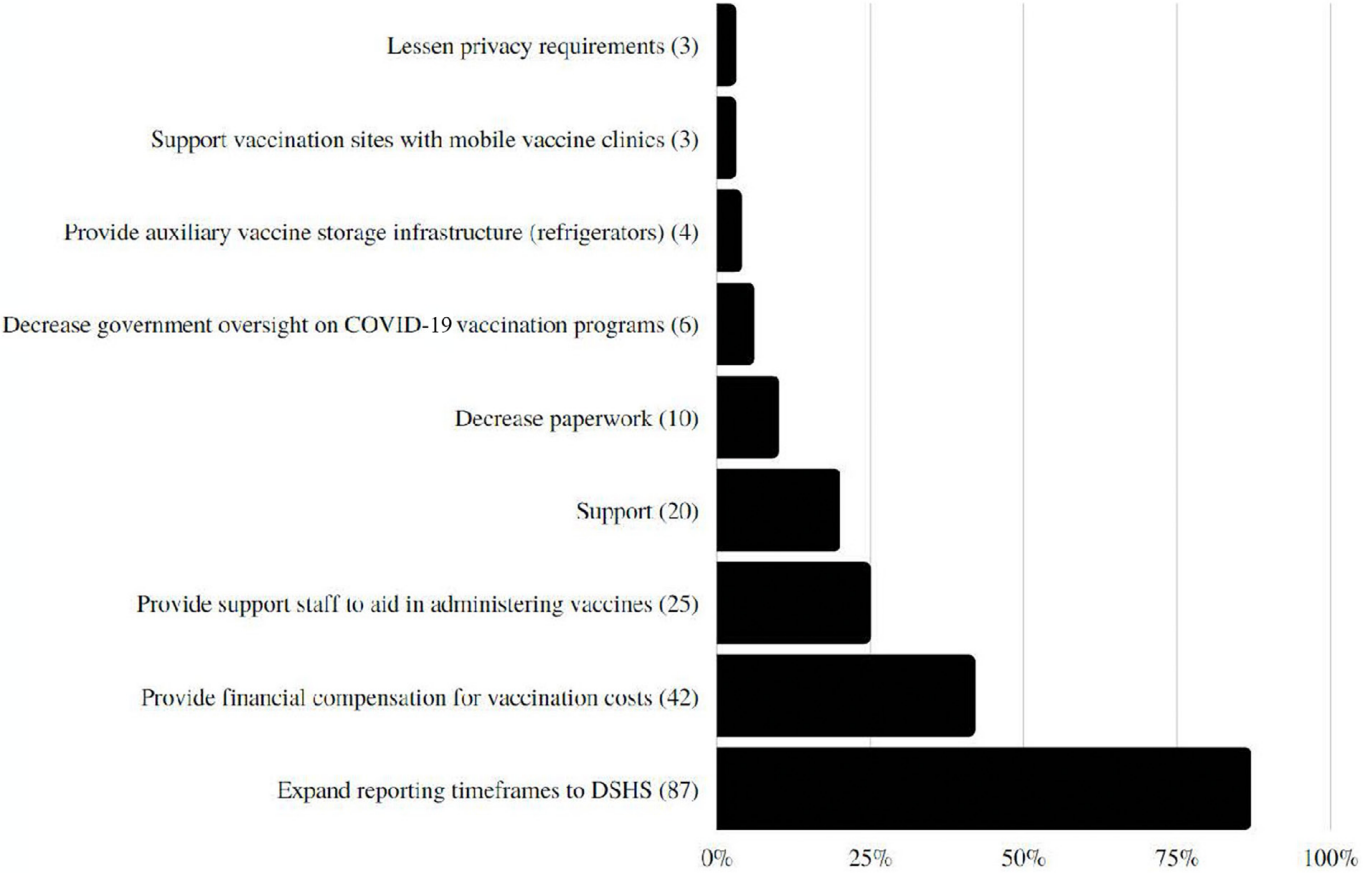
Provider recommendations on how SAMHD can aid physicians’ vaccination efforts.

DSHS, Department of State Health Services; SAMHD, San Antonio Metropolitan Health District.

Respondents were also asked about how SAMHD could aid in physicians’ future vaccination efforts (Figure 2). Physicians commonly requested some type of financial compensation (*n*=30, 42%) to alleviate the costs of vaccination as well as increasing the 24-hour reporting timeframe for entry of confirmed COVID-19 vaccination into the Texas Department of State Health Services (DSHS) vaccine registry database (*n*=35, 87%). Additional recommendations included providing trained support staff (*n*=18, 25%) to aid in administering vaccines as well as improving logistical support (*n*=14, 20%), targeting the medical infrastructure to store and administer vaccines.

## DISCUSSION

The goal of this initiative was to improve provider participation in the vaccine rollout, support providers in their vaccine administration programs, and combat vaccine hesitancy. To address provider concerns and respond to their recommendations, survey results were shared with SAMHD vaccination leaders and members of the local COVID-19 Community Response and Equity Coalition (CREC) who were engaged in vaccination efforts. These recommendations helped to guide vaccine logistical planning and education for the general Bexar County population as well as healthcare providers. This was especially pertinent considering the global impact of the COVID-19 pandemic. With more than 220 million cases worldwide, the economic ramifications, caused by loss of industrial production and general health reduction, have been upward of 16 trillion dollars.^11^ It was evident that finding a cure to COVID-19 to curb deaths and developing a vaccine to minimize its spread as fast as possible were the primary goals worldwide.

In the early stages of vaccine development, the efficacy of the vaccine was uncertain, but it was estimated that coverage of 75% of the population would need to be vaccinated with an assumed efficacy of 70%.^12^ However, reports from Phase 3 RCTs showed a 95% efficacy, with estimated arrival times of vaccines being as early as December 2020.^13^ This expedited delivery was possible because the vaccines were administered under EUA. EUA is a program that allows for the use of medical products to prevent serious diseases or conditions, such as COVID-19, in response to public health emergencies. This occurs when the risk of not treating the problem is deemed more serious than the potential risk of the new product. In this case, the vaccine is still tested for its safety and efficacy.^14^ After issuing the EUA, FDA continued to monitor the vaccines, and on August 23, 2021, the Pfizer vaccine gained full FDA approval.^15^ Despite this, vaccine hesitancy began to grow in the general population because of messenger RNA vaccines being introduced as a novel method for mass immunization during a public health emergency and the expedited development with heavy involvement of government agencies. The factors described earlier led to the rapid spread of information and misinformation, contributing to today's vaccine hesitancy.

Vaccine hesitancy is an ever-evolving issue, and it is important to correlate and compare findings not only between different points in time but also between different groups of people. A study by Biswas et al.^16^ published in 2021 stated that, among the general population of the U.S., about 32% reported some degree of vaccine hesitancy. A similar survey conducted by Lucia and colleagues^17^ stated that 23% of medical students would not take the vaccine immediately after approval, whereas one by Paris et al showed that <60% of auxiliary nurses and technicians intended to be vaccinated, as compared with 60%–79% of nurses and support staff, and >80% of medical staff. Arce and colleagues^18^ found an increased rate of vaccine hesitancy in the U.S. (35%) compared with that in lower-/middle-income countries (20%).^17^^,^^18^ Finally, a study of healthcare workers showed a hesitancy rate of 28%.^19^ With hesitancy among these populations in context, especially within the physician population, this project aimed to understand physician and provider sentiments regarding the vaccine and its administration to the respective patient populations they care for, ultimately gathering their feedback to best prepare for the vaccine rollout. The information collected was then used to improve messaging and address common concerns of providers.

Representing most ZIP codes in Bexar County, survey responses were well representative of the Bexar County primary care vaccine provider population. Nullifying concerns before the survey release, provider responses to whether they would recommend the vaccine showed that 20% of respondents supported the administration of vaccines only under FDA full licensure approval, whereas 77% were willing to support administration under EUA. Only 3% of respondents were completely opposed to vaccinating their patient populations. Most provider concerns were related to the expedited development of vaccines, distrust of government agencies, and logistical barriers associated with vaccine storage and vaccination costs. Moreover, to address vaccine hesitancy, providers suggested improving information delivery of vaccine-related information through education programs that present standardized information in a transparent, unbiased, and factual manner. All these variables were considered by SAMHD and the CREC in designing initiatives to aid vaccination programs and combat vaccine hesitancy.

In efforts to promote participation, the BCMS provided information on other Bexar County physicians who did not participate in the study. Geographic gaps were identified in ZIP codes where there were a low number of providers offering vaccines. After these gaps were determined, community health and prevention teams consisting of members from Metro Health, STRAC, San Antonio Police Department, the San Antonio Fire Department, and other city departments set out on foot to canvass providers in these ZIP codes. These teams went door to door to initiate direct contact with physicians in these areas to raise awareness and recruit them to enroll in physician vaccination networks. These canvassing efforts were conducted between January 2021 and February 2021, resulting in the registration of an additional 150 vaccine providers.

Survey data were also used to augment existing vaccine administration programs. Providers expressed concern regarding difficulties with DSHS protocols for reporting vaccination data. Primarily, the 24-hour timeframe for reporting vaccination data was seen as unreasonable. Many providers were also unsatisfied with the DSHS reporting interface. Although reporting protocols for vaccination data were not changed, the DSHS did respond to these concerns by updating its website's interface to be more user friendly. In doing so, the administrative aspects of conducting a vaccination program were facilitated, encouraging more providers to engage in the vaccination efforts. In addition, several providers raised concerns about logistical challenges related to vaccine storage, lack of personnel to engage in vaccination efforts, and financial strains that limited them from providing vaccinations. To remedy this, several providers recommended the implementation of pop-up clinics to support areas with low densities of vaccine providers. SAMHD and CREC collaboratively responded to these tasks by organizing pop-up clinics in ZIP codes with low numbers of registered vaccine providers. As of October 2, 2021, these pop-up clinics have administered more than 24,700 doses of the COVID-19 vaccine, fully vaccinating more than 12,900 Bexar County residents. This support from SAMHD has allowed for more providers to handle the obligations of a vaccination effort at their respective healthcare facilities and increase vaccination rates in Bexar County.

Based on the results of this study, it was understood that there were still gaps in physician knowledge of the vaccine and COVID-19 in general. Thus, SAMHD followed provider recommendations by implementing vaccine education programs for physicians. Provider education and outreach were tailored to address issues such as vaccine storage requirements and storage capacity while promoting vaccinations within provider networks. An evidence-based medicine team was also established, consisting of medical students and faculty advisors from the Long School of Medicine at UT Health San Antonio. This team has published monthly literature reviews about the epidemiology, diagnostics, clinical presentation, and treatment of COVID-19, creating executive summaries and concise information sheets to better inform and educate clinicians on the latest evidence-based updates regarding various aspects of the COVID-19 pandemic.^20^

### Limitations

This study was designed to gather Bexar County physicians' sentiments toward the vaccine and allow them to articulate potential issues regarding the vaccine rollout. However, it was not designed to measure the impact of subsequent initiatives conducted to address provider issues or aid vaccine rollout. In addition, the study was exclusively administered to healthcare providers serving Bexar County residents, limiting our ability to generalize to other physicians in Texas and beyond. In addition, our use of professional networks (e.g., STRAC and BCMS) and Metro Health physician listservs meant that we could not establish a full sampling frame of potential providers and are therefore unable to determine the representativeness of our study sample. We believe that this was an appropriate sacrifice considering the emergent need for this study to inform our collective public health response. During analysis, we also identified gaps in responses within some ZIP codes in Bexar County. This trend was reflective of the SES of the patient populations: the lower the status, the fewer responses from physicians, which we know is due in part to a lack of healthcare providers serving people in those ZIP codes as well as the incomplete sampling frame mentioned earlier. However, it should be noted that Metro Health used these results to canvas the underrepresented ZIP codes to identify and support physicians working in those ZIP codes. Finally, the optional nature of some of the questions resulted in lower sample sizes. Because of this, the study has its limitations, but it provides significant insight into providers’ sentiments and valuable suggestions to optimize future vaccination endeavors.

## CONCLUSIONS

With vaccine hesitancy continuing to be a significant issue related to COVID-19 and future vaccine rollouts, it is important to understand the sentiments of a region's provider population and work with them to establish initiatives targeted toward combating sources of hesitancy and aiding vaccination efforts. The outcomes of this study and the efforts of SAMHD and the CREC can be replicated in other populations to help improve the efficiency of vaccination programs and reduce vaccine hesitancy.

## Appendix Supplementary materials

Image, application 1
