# Supplementary material for: Understanding and Addressing COVID-19 Vaccine Hesitancy Among Healthcare Providers in Bexar County, Texas
Source: AJPM Focus. 2022 Aug 10;1(2):100022. doi: 10.1016/j.focus.2022.100022 (PMC9364726; doi:10.1016/j.focus.2022.100022)
Supplement: Supplementary file 1 [file mmc1.pdf]

Covid19 Vaccine Provider Survey for San Antonio

Dear Prospective Participant,

The success of the COVID-19 vaccination campaign will depend on the collaboration of health care providers to administer pandemic vaccines. In collaboration with the COVID-19 Community Response Coalition (CRC), UT Health San Antonio and San Antonio Metropolitan Health District (Metro Health) will utilize a survey to assess provider willingness to serve as a COVID-19 vaccine provider. The results will help Metro Health form policies for implementation and administration of vaccination, once available.

What are the key details when deciding to volunteer to contribute to the study?

Your participation in this survey as a healthcare professional working in any healthcare setting, and actively dealing with the current COVID-19 pandemic, is completely voluntary. The results from this research study will help us understand your concerns, your perspective, your understanding of the vaccine development process and its implementation to mitigate this healthcare crisis. This survey will also help the medical community and administrative personnel to form policies for implementation and administration of vaccination, once available.

Although you may not get personal benefit from taking part in this research study, your responses may help the medical community and administrative authorities to help address concerns about the upcoming COVID-19 vaccination and its implementation.

What are the key details when deciding not to volunteer to contribute to the study?

Participation in this research study is completely voluntary and no personally identifiable data will be released. If you choose not to participate in this survey close the browser at any time, before the final submission. Your response to the survey will be kept confidential to the extent allowed by the law. When we write about the study and its results you will not be identified.

This is a non-funded research study and there is no compensation for taking part in this study.The survey will take 5-10 minutes to complete.There are no known risks to participate in this study. Although we have tried to minimize this, some questions may make you upset, or make you feel uncomfortable, and you may choose not to answer them.All survey responses are stored in a password protected electronic format. The final results of the survey will be shared with all of the community partners, and with you.

The University of Texas Health Science Center is the local Institutional Review Board committee that reviews research on human subjects (Institutional Review Board) and can also answer any questions about your rights as a research subject, and take any concerns, comments or complaints you may wish to offer.

You can contact the IRB by calling 210-567-8250, or by mail to IRB, UTH San Antonio, Mail Code 7830, 7703 Floyd Curl Drive, San Antonio, TX 78229-3900.

Thank you in advance for your assistance with this important project. To ensure your responses are included in the study, please complete your survey by December 7, 2020.

Consent to participate

Choosing AGREE indicates that:

You have read the above information You have been emailed a copy of the survey information You voluntarily agree to participate You are at least 18 years of age

If you do not wish to participate in the survey, please decline participation by choosing DISAGREE.

○ AGREE
○ DISAGREE

Are you conducting a proxy interview? If so, please enter your name in this field and the provider information throughout the survey.

____

Healthcare provider information

Name ______________________________

Email Address ______________________________

Work Phone ______________________________

Zip code where you practice ______________________________

Healthcare provider type (please select 1):

- ◯ Private primary care
- ◯ Private specialty care
- ◯ Urgent care/emergency care
- ◯ Occupational health clinic
- ◯ Private hospital
- ◯ Long-term care
- ◯ Federally Qualified Health Center (FQHC)
- ◯ Government clinic/hospital

Population(s) routinely served

	Yes	No	I don't know
Children 18 years of age and younger	◯	◯	◯
Adults 19-64 years of age	◯	◯	◯
Adults 65 years of age and older	◯	◯	◯
Long term care facility residents (nursing home, assisted living, or independent living facility)	◯	◯	◯
Health care workers	◯	◯	◯
Military-active duty/reserves	◯	◯	◯
Military-veteran	◯	◯	◯
People experiencing homelessness	◯	◯	◯
Pregnant women	◯	◯	◯
Racial and ethnic minority groups	◯	◯	◯
People who are under-insured or uninsured	◯	◯	◯

On average, how many unique patients are seen each month? ______________________________

Organization name

Organization name

____

Organization telephone

____

Organization zip code

____

About your organization

	Yes	No	I don't know
Does this organization participate in the Vaccines for Children (VFC) program?	○	○	○
Does this organization participate in the Adult Safety Net (ASN) program?	○	○	○
Does this organization accept Medicaid?	○	○	○

Vaccine storage and handling

Does your facility have the capability to store vaccine at the following temperature? Check all that apply.

- ☐ Refrigerated (2 to 8 C / 36 to 46 F)
- ☐ Frozen (-15 to -25 C / 5 to -13 F)
- ☐ Ultra-frozen (-60 to -80 C / -76 to -112 F)

Provider perspectives

What are the key sources you use to inform your opinion on prospective COVID-19 vaccines? Check all that apply.

- ☐ Published and/or publicly available clinical trial data
- ☐ Governmental agencies (CDC, EMA, State & Local Health department etc.)
- ☐ Key opinion leaders in infectious diseases field
- ☐ Global/state-wide physician networks (Texas Medical Association)
- ☐ Press articles summarizing clinical trial data
- ☐ Local physician networks (Bexar County Medical Society)
- ☐ Key opinion leaders in other field
- ☐ Personal experience with vaccines and/or COVID-19
- ☐ Pharmaceutical companies
- ☐ Discussions with your patients
- ☐ Social media
- ☐ Non-governmental agencies
- ☐ Other sources

If you selected "other," please specify:

____

Which of the following would be important for you in making the decision to get vaccinated for COVID-19?

	Strongly disagree	Disagree	Neutral	Agree	Strongly agree
I am worried about the rapidity of the development and approval of COVID-19 vaccine	◯	◯	◯	◯	◯
I do not trust the pharmaceutical companies developing/manufacturing the vaccine	◯	◯	◯	◯	◯
I do not trust the FDA overseeing the vaccine development/safety and might have political motivation for rapid approval	◯	◯	◯	◯	◯
I do not trust the CDC overseeing the vaccine development/safety and might have political motivation for rapid approval	◯	◯	◯	◯	◯
I do not trust the information provided by the federal government about COVID-19 or its severity	◯	◯	◯	◯	◯

What are your biggest concerns right now about potential COVID-19 vaccines?

____

For health care workers, do you think the COVID-19 vaccine should be:

◯ Voluntary
◯ Mandated by the employer, like Influenza vaccine
◯ Mandated by the State government for all health care workers
◯ Mandated by the Federal government for all health care workers
◯ Not sure

For adult patients in your practice(s), what do you think will be the main barriers to adoption for a COVID-19 vaccine?

- ☐ Belief that they wouldn't get the disease and/or the symptoms would be mild
- ☐ They don't like people/government telling them what to do
- ☐ Concern about the vaccine safety and potential side-effects
- ☐ Belief that the vaccine is not efficacious and hence not worth the hassle
- ☐ Concern about their ability to afford the vaccine
- ☐ Concern about their ability to access a site of administration
- ☐ Religious beliefs and/or cultural beliefs
- ☐ They forgot to get vaccinated or were too busy
- ☐ They had a medical condition that prevents vaccination (e.g., patient is immunocompromised)
- ☐ They did not know a vaccine was available
- ☐ Fear of needles
- ☐ They do not trust the regulatory authorities (FDA/CDC) overseeing the vaccine development/safety and might have political motivation for rapid approval
- ☐ They do not trust the information provided by the federal government about COVID-19 or its severity
- ☐ They do not trust the pharmaceutical companies developing/manufacturing the vaccine
- ☐ They believe myths about the COVID-19 vaccine
- ☐ Other reason

Other reason ______________________________

How might Metro Health and the COVID-19 CRC support your vaccine education and outreach efforts in addressing the patient barriers you identified in the previous question? ______________________________

The Food and Drug Administration issued guidance with recommendations for vaccine sponsors regarding issuance of an emergency use authorization (EUA) for an investigational vaccine intended to prevent COVID-19. An EUA is a different standard than an approval, however, both pathways require the submission of data demonstrating any vaccine's safety and effectiveness.

Under which approval conditions would you be willing to recommend a COVID-19 vaccine to the following patient populations?

	Under an Emergency Use Authorization (EUA)	Under an FDA full licensure approval	Either	Neither
Adults 65 years and older	○	○	○	○
Adults 18-65 years old without comorbidities	○	○	○	○

Adults with severe comorbidities (e.g. cancer)	◯	◯	◯	◯
Adults with moderate comorbidities (e.g. obesity)	◯	◯	◯	◯
Adults with occupational risk (e.g. healthcare workers)	◯	◯	◯	◯
Pediatrics (under 18 years old)	◯	◯	◯	◯

What could lead you to recommend an approved COVID-19 vaccine earlier for your patients? Check all that apply.

- ☐ More robust safety data
- ☐ Worsening of COVID-19 outbreak in my region
- ☐ Broad acceptance of the vaccine by the healthcare community
- ☐ More robust efficacy data
- ☐ Broad acceptance of the vaccine by the public
- ☐ Other reasons

Other reasons that would lead you to recommend an approved vaccine:

____

Metro Health is building partnerships within community stakeholders to spearhead COVID-19 immunization initiatives to improve vaccination rates and is exploring an on-site mobile clinic option to provide services.

What benefits do mobile on-site vaccinations provide?
Convenience - We can vaccinate all your patients collectively, without them having to travel
Compliance - Our vaccinations, medical staff, and online portal remain entirely compliant with medical regulations Professional - Our skilled team of medical practitioners will efficiently and safely perform vaccinations

Would your organization be interested in hosting a COVID-19 vaccine mobile clinic for your patients?

- ☐ Yes, if we could meet the requirements
- ☐ I need more information
- ☐ Not at this time

Do you have the space for a 40-foot long, 14-foot high mobile clinic?

- ☐ Yes
- ☐ I don't know
- ☐ No

The CDC and DSHS require that organizations enrolled in the COVID-19 Vaccination Program report data elements for each dose administered. Additional requirements for COVID-19 Vaccine Administration are below.

How likely are you to participate under these potential requirements?

	Extremely unlikely	Unlikely	Neutral	Likely	Extremely likely

Administer COVID-19 Vaccine regardless of the vaccine recipient's ability to pay COVID-19 Vaccine administration fees.	○	○	○	○	○
Report the number of doses of COVID-19 Vaccine and adjuvants that were unused, spoiled, expired, or wasted as required by DSHS.	○	○	○	○	○
Not sell or seek reimbursement for COVID-19 vaccine and any adjuvant, syringes, needles, or other constituent products and ancillary supplies that the federal government provides without cost to the Organization.	○	○	○	○	○
Organization must comply with all federal instructions and timelines for disposing COVID-19 vaccine and adjuvant, including unused doses.	○	○	○	○	○
Organization's COVID-19 vaccination services must be conducted in compliance with CDC's Guidance for Immunization Services During the COVID-19 Pandemic for safe delivery of vaccines.	○	○	○	○	○
Organizations report online data elements for each dose administered within 24 hours of administration to the Texas Immunization Registry, ImmTac2.	○	○	○	○	○

If all of these requirements remain in force, what could be done that would motivate you to participate?

____

What requirements, if removed, would make it likely that you would participate?

____

Are you willing to answer a few demographic questions for our research?

○ Yes
○ No

Gender

○ Female
○ Male
○ Non-binary/not specified above
○ Do not wish to answer

Ethnicity	◯ Hispanic or Latino<br>◯ Not Hispanic or Latino<br>◯ Unknown/Not reported<br>◯ Do not wish to answer
Race	◯ Native Americans/Alaska Native<br>◯ Asian<br>◯ Native Hawaiian or Other Pacific Islander<br>◯ Black or African American<br>◯ White or Caucasian<br>◯ More Than One Race<br>◯ Unknown / Other<br>◯ Do not wish to answer
How many years have you practiced post-residency/training?	◯ Less than 10 years<br>◯ 10-20 years<br>◯ More than 20 years<br>◯ Do not wish to answer
